# Raf/ERK/Nrf2 signaling pathway and MMP-7 expression involvement in the trigonelline-mediated inhibition of hepatocarcinoma cell migration

**DOI:** 10.3402/fnr.v59.29884

**Published:** 2015-12-22

**Authors:** Jung Chun Liao, Kun Tsung Lee, Bang Jau You, Chia Lin Lee, Wen Te Chang, Yang Chang Wu, Hong-Zin Lee

**Affiliations:** 1 School of Pharmacy, China Medical University, Taichung, Taiwan; 2 Department of Oral Hygiene, College of Dental Medicine, Kaohsiung Medical University, Kaohsiung, Taiwan; 3 School of Chinese Pharmaceutical Sciences and Chinese Medicine Resources, China Medical University, Taichung, Taiwan; 4 Department of Cosmeceutics and Graduate Institute of Cosmeceutics, China Medical University, Taichung, Taiwan; 5 Pharmacy Department, Tainan Municipal An-Nan Hospital-China Medical University, Tainan, Taiwan

**Keywords:** trigonelline, hepatocarcinoma Hep3B cells, migration, *Pisum sativum*, chemotherapeutic agent, Raf/ERK/Nrf2 signaling pathway, MMP-7

## Abstract

**Background:**

Trigonelline occurs in many dietary food plants and has been found to have anti-carcinogenic activity. Trigonelline is also found in coffee which *is one of the most* widely *consumed beverages*. Many epidemiological studies have reported that coffee consumption has an inverse relationship with the risk of cirrhosis or hepatocellular carcinoma. It would be interesting to investigate whether trigonelline is an ideal chemoprevent agent to prevent cancer progression.

**Methods:**

The protein expression was performed by western blotting. The trigonelline content in snow pea (*Pisum sativum*) was analyzed by high-performance liquid chromatography (HPLC). The migratory activity of human hepatocarcinoma cells (Hep3B) was assessed by using a wound migration assay. The percentage of each phase in the cell cycle was analyzed on a FACScan flow cytometer. Gene expression was detected by real-time reverse transcriptase-polymerase chain reaction techniques. Native gel analysis was performed to analyze the activity of superoxide dismutase (SOD), catalase and glutathione peroxidase.

**Results:**

According to the data of HPLC analysis, *P. sativum*, which is a popular vegetable, has relatively high content of trigonelline. Our findings suggest that trigonelline is an efficient compound for inhibiting Hep3B cell migration. Trigonelline inhibited the migration of hepatoma cells at concentrations of 75–100 µM without affecting proliferation. Raf/ERK/Nrf2 protein levels and further downstream antioxidative enzymes activity, such as SOD, catalase, and glutathione peroxidase, significantly decreased after treatment with 100 µM of trigonelline for 24 h. The migration inhibition of trigonelline is also related to its ability to regulate the matrix metalloproteinases 7 (MMP-7) gene expression.

**Conclusions:**

In this study, protein kinase Cα (PKCα) and Raf/ERK/Nrf2 signaling pathway and MMP-7 gene expression were involved in the trigonelline-mediated migration inhibition of Hep3B cells. We also demonstrated that trigonelline inhibits Hep3B cell migration through downregulation of nuclear factor E2-related factor 2–dependent antioxidant enzymes activity. This study analyzed the trigonelline content in a popular vegetable, snow pea, as a representative proof to prove that trigonelline is often found in the daily intake of food. Our finding suggested that trigonelline should be a useful chemopreventive agent derived from the daily intake of food to prevent cancer progression.

Trigonelline is an alkaloid and occurs in many plants, such as fenugreek seeds, garden peas, and coffee (1–5). Coffee is one of the most widely consumed beverages. Furthermore, the coffee drinking population increased rapidly in the past decade. Coffee is rich in many bioactive substances and its consumption has been associated with many beneficial effects. Four representative components of coffee's micronutrients, namely, caffeine, chlorogenic acid, diterpenes, and trigonelline, play key roles in the bioactive effects of coffee (1). Quantitative analysis of the bioactives trigonelline in a large set of roasted Arabica and Robusta coffees revealed a high trigonelline content in coffee beans (2, 3). Coffee is believed to have an anti-carcinogenic activity, anti-thrombotic properties, and antioxidant activity via interfering with nuclear factor E2-related factor 2 (Nrf2) activation (1, 6, 7). According to the report provided by the Ministry of Health and Welfare in Taiwan, liver cancer was the second leading cause of death in 2013. Since liver cancer presents a serious public health problem and economic burdens on the society, many investigators have exerted efforts to prevent the development of the cancer. Many epidemiological studies have reported that coffee consumption has an inverse relationship with the risk of cirrhosis or hepatocellular carcinoma (8, 9). Furthermore, Hirakawa et al. (10) suggested that trigonelline inhibits the invasion of hepatoma cells without affecting the proliferation of the cells. Although trigonelline has been found to have anti-carcinogenic effects, there is no substantial evidence relating trigonelline to treatment of cancer. In this study, trigonelline was investigated for its anti-invasive and chemopreventive activity in hepatocarcinoma cells.

Nrf2 is a transcription factor that plays a critical role in coordinated induction of genes encoding numerous phase II detoxifying and antioxidant enzymes, such as superoxide dismutase (SOD), catalase, and heme oxygenase-1 (11, 12). Since Nrf2 is a primary cellular defense protein against the cytotoxic effects of oxidative stress, Nrf2 pathway was considered as an important event in cancer cell proliferation and tumorigenesis. Recently, many chemopreventive compounds, such as sulforaphane, curcumin, and resveratrol, have been proven to be able to prevent tumorigenesis through activation of Nrf2 and further downstream antioxidant genes (13–15). Furthermore, Sebens et al. (16) have suggested that loss of Nrf2 through targeted gene deletion decreases cell survival in response to oxidative stress. However, a great number of malignant tumors, including colon, lung, breast, and pancreatic cancer, exhibit an increased activity of Nrf2 (17–20). Nrf2 phosphorylation is a key event in the regulation of Nrf2-mediated antioxidant response, providing for its activation (21, 22). In addition, activation of the Raf/ERK signaling cascade in human cancer cells has been demonstrated to be required for Nrf2 activation, which promotes Nrf2 nuclear translocation and binding to the specific DNA sequence (23, 24).

Matrix metalloproteinases (MMPs) are zinc-dependent endopeptidases with the ability to degrade extracellular matrix proteins and play a fundamental role in inflammation, tissue remodelling, angiogenesis, wound healing, tumor invasion, and metastatic progression. MMP-2 and -9 are the kind of gelatinase and collagenase demonstrated to be involved in migration and invasion of tumor cells (25, 26). MMP-7, also called matrilysin, is capable of degrading many cellular adhesions proteins and plays a role in tumor progression. MMP-7 is often overexpressed in a variety of malignant tumors including colon cancer, gastric carcinoma, and squamous cell carcinoma of tongue and oral cavity (26–28). Recent studies have demonstrated that the expression of Nrf2, in addition to its antioxidative effects, was involved in the MMP activity and cancer cell invasion and migration (29, 30).

An ideal chemopreventive agent should have few or no toxic effects and the potential to reverse, suppress, or prevent carcinogenic progression to invasive cancer. The aim of this study was to examine whether trigonelline which occurs in many dietary food plants can inhibit liver cancer cell migration. This study also characterized the effect of trigonelline on the gene expression of MMP-2, -7, and -9. Since the expression of Nrf2 plays an important role in regulating MMP activity, this study characterized the expression of Raf/ERK/Nrf2 signaling pathway in human hepatocarcinoma cells (Hep3B) exposed to trigonelline.

## Materials and methods

### Materials

The voucher specimens (*Pisum sativum* L. var. *saccharatum* Poir: CMU-104-PS-003) were deposited in Herbarium of College of Pharmacy, China Medical University, Taichung, Taiwan. Antipain, aprotinin, dithiothreitol, ethyleneglycol-*bis*(*β*-aminoethyl ether)-*N,N,N′,N′*-tetraacetic acid, leupeptin, Nonidet P-40, pepstatin, phenylmethylsulfonyl fluoride, sodium deoxycholate, trigonelline, and 2-amino-2-hydroxymethyl-propane-1,3-diol (Tris) were purchased from Sigma Chemical Company (St. Louis, MO). Antibodies to various proteins were obtained from the following sources: β-actin antibody was purchased from Sigma Chemical Company; Caspase-9, catalase, p38 (pThr180/Tyr182), and Raf (pSer259) were purchased from Abcam (Cambridge, MA); Mn-SOD and Cu/Zn-SOD were from Calbiochem (San Diego, CA); ERK (pThr202/Tyr204) was from ThermoFisher Scientific, Inc. (Waltham, MA); Nrf2 (pSer40) was from GeneTex, Inc. (Irvine, CA); Caspase-3 and protein kinase Cα (PKCα) from BD Biosciences (San Jose, CA). Horseradish peroxidase (HRP)-conjugated goat anti-mouse and -rabbit IgG were from Abcam.

### Preparation and fractionation of snow pea (*Pisum sativum* L. var. saccharatum Poir)

The botanical origin of *P. sativum* was identified by Dr. Chao-Lin Kuo (School of Chinese Pharmaceutical Sciences and Chinese Medicine Resources, China Medical University, Taichung, Taiwan). The plants of *P. sativum* (600 g) were soaked four times with 1 L of 95% ethanol at room temperature for 3 days. The crude extracts were concentrated under reduced pressure at 40°C. The ethanol extracts (45.5 g) were partitioned between ethyl acetate and H_2_O (1:1, v/v) to give an ethyl acetate soluble fraction (3.7 g) and an aqueous phase (31.8 g), which were then partitioned with *n*-hexane/95% methanol (1:1) and *n*-butanol/H_2_O (1:1), respectively, to give four fractions. The ethanol extracts of *P. sativum* were partitioned into *n*-hexane (0.89 g), 95% methanol (2.25 g), *n*-butanol (27.1 g), and H_2_O (13.71 g) soluble fractions, using an initial ethyl acetate and H_2_O extract.

### High-performance liquid chromatography assay for *P. sativum* fractions

High-performance liquid chromatography (HPLC) was performed by an Inertsil ODS-3V column (5 µm, 4.6×150 mm, GL Science, Inc., Tokyo, Japan) eluted at a rate of 1.0 ml/min with a mobile phase of 0.1% formic acid solution and acetonitrile (95/5, v/v) and UV detector with the detection wavelength set at 267 nm. All samples dissolved in methanol were filtered through 0.45 µm Millipore membrane prior to HPLC analysis. The injection volume was 10 µl. To quantify trigonelline in the fractions of *P. sativum*, the standard curve of trigonelline was made in a series of concentrations in the ranges of 5.0–300.0 µg/ml. Calibration graphs were plotted by linear regression analysis of the peak area with concentrations. For qualitative analysis of trigonelline in the fractions of *P. sativum*, 15 µg/ml trigonelline was added to the fraction extracts of *P. sativum*.

### Human hepatocellular carcinoma cell line Hep3B

The human hepatocellular carcinoma cell line Hep3B was kindly provided by Professor Yang-Chang Wu (China Medical University, Taichung, Taiwan). Hep3B cells were grown in monolayer culture in Dulbecco's modified Eagle's medium (Life Technologies, Rockville, MD) containing 5% fetal bovine serum (HyClone, Logan, UT), 100 U/ml penicillin, 100 µg/ml streptomycin, and 2 mM glutamine at 37°C in a humidified atmosphere comprising 95% air and 5% CO_2_.

### Mitochondrial reductase activity assay

Cells were seeded at a density of 2.8×10^4^ cells per well onto a 12-well plate 48 h before being treated. After treatment, cellular mitochondrial reductase activity of live Hep3B cells was detected by measuring the reduction of 3-(4,5-dimethylthiazol-2-yl)-2,5-diphenyltetrazolium bromide (MTT).

### Flow cytometric analysis

The percentage of each phase in the cell cycle was determined as described previously (31). Briefly, cells were collected and fixed in 80% ethanol. Fixed cells were incubated with 100 µg/ml RNase A, stained with propidium iodide (50 µg/ml), and analyzed on a FACScan flow cytometer (Becton Dickinson Instruments).

### Wound healing assay

The migratory activity of Hep3B cells was assessed using a wounded migration assay. Cells were seeded at a density of 5×10^4^ cells/well onto 12-well plates and cultured for 48 h until they reached confluence or near confluence. A linear wound was made by scratching the monolayer with a sterile (yellow) pipette tip. The scratch width of the wound cell without incubation was about 248.1±10.6 µm (time point 0 h). After washing, cells were supplied with 1 ml complete medium in the absence (control) or presence of different concentrations of trigonelline. After 48-h incubation, wounded areas were photographed with an Olympus IX 70 phase-contrast microscope (Olympus Optical Co., Tokyo, Japan).

### Evaluation of SOD, catalase, and glutathione peroxidase activity

The activity of SOD, catalase, and glutathione peroxidase was evaluated as previously described (32, 33). Adherent and floating cells were collected and sonicated in cold 50 mM phosphate buffer. The protein concentrations were estimated with the Bradford method. Electrophoresis was performed in 10% polyacrylamide gels without 0.1% sodium dodecyl sulfate (SDS). To examine SOD activity, the polyacrylamide gel was then stained with nitroblue tetrazolium (33). To evaluate catalase activity, the gel was detected following a 5-min treatment in 5% methanol, three water rinses, 5 min incubation in 0.03% H_2_O_2_, and incubation in 1% ferric chloride and 1% potassium ferricyanide solution (33). For detecting glutathione peroxidase activity, the gel was submerged in a 50 mM Tris–HCl buffer (pH 7.9) containing 13 mM glutathione and 0.004% hydrogen peroxide with gentle shaking for 20 min. The glutathione peroxidase activity was stained by 1.2 mM MTT and 1.6 mM phenazine methosulfate (32). The activity band showed a clear zone against blue backgrounds.

### Real-time reverse transcriptase-polymerase chain reaction

Total RNA was extracted by using MagNA Pure Compact RNA Isolation Kit (Roche Applied Science, Indianapolis, IN). The quantity of RNA samples was determined using NanoDrop ND-1000 (ThermoFisher Scientific). RNA samples were reverse transcribed for 120 min at 37°C with High Capacity cDNA Reverse Transcription Kit according to the standard protocol of the supplier (Applied Biosystems, Foster City, CA). Quantitative PCR was performed under the following condition: 10 min at 95°C, 40 cycles of 15 sec at 95°C, and 1 min at 60°C using 2× Power SYBR Green PCR Master Mix (Applied Biosystems) and 200 nM of forward and reverse primers. The primer sequence for real-time reverse transcriptase-polymerase chain reaction (RT-PCR) was 5′-CCCCAGACAGGTGATCTTGAC-3′ and 5′-GCTTGCGAGGGAAGAAGTTG-3′ for MMP-2; 5′-GGATGGTAGCAGTCTAGGGATTAACT-3′ and 5′-AGGTTGGATACATCACTGCATTAGG-3′ for MMP-7; 5′-CGCTGGGCTTAGATCATTCC-3′ and 5′-GTGCCGGATGCCATTCAC-3′ for MMP-9; and 5′-ACACCCACTCCTCCACCTTT-3′ and 5′-TAGCCAAATTCGTTGTCATACC-3′ for glyceraldehyde 3-phosphate dehydrogenase (GAPDH) (34). For data analysis, the comparative threshold cycle (*C*_T_) method was used. Each assay was run on an Applied Biosystems 7300 Real-Time PCR system in triplicates and expression fold-changes were derived using the comparative *C*_T_ method. Results were the average relative mRNA expression of the different genes normalized to GAPDH.

### Protein preparation and western blot analysis

Protein preparation and western blot analysis were performed as previously described (31). After electrophoresis, the SDS-separated proteins were electrotransferred to PVDF membranes (Millipore) and then probed with antibodies to β-actin (1:1,000), caspase-3 (1:1,000), caspase-9 (1:200), catalase (1:2,000), Cu/Zn-SOD (1:25,000), ERK (pThr202/Tyr204) (1:2,000), Mn-SOD (1:4,000), Nrf2 (pSer40) (1:8,000), p38 (pThr180/Tyr182) (1:1,000), PKCα (1:1,000), and c-Raf (pSer259) (1:1,000). Secondary antibody consisted of a 1:20,000 dilution of HRP-conjugated goat anti-mouse IgG (for caspase-3, Mn-SOD, and PKCα), HRP-conjugated rabbit anti-sheep IgG (for Cu/Zn-SOD), or HRP-conjugated goat anti-rabbit IgG [for caspase-9, catalase, ERK (pThr202/Tyr204), Nrf2 (pSer40), p38 (pThr180/Tyr182), and Raf (pSer259)].

### Data analysis and statistics

Values are presented as percentage±SD of control. Statistically significant difference from the control group was identified by Student's *t*-test for paired data. A *P*-value <0.05 was considered significant for all tests.

## Results

### Trigonelline is the constituent of snow pea (*Pisum sativum* L. var. saccharatum Poir)

**Fig. 1:**
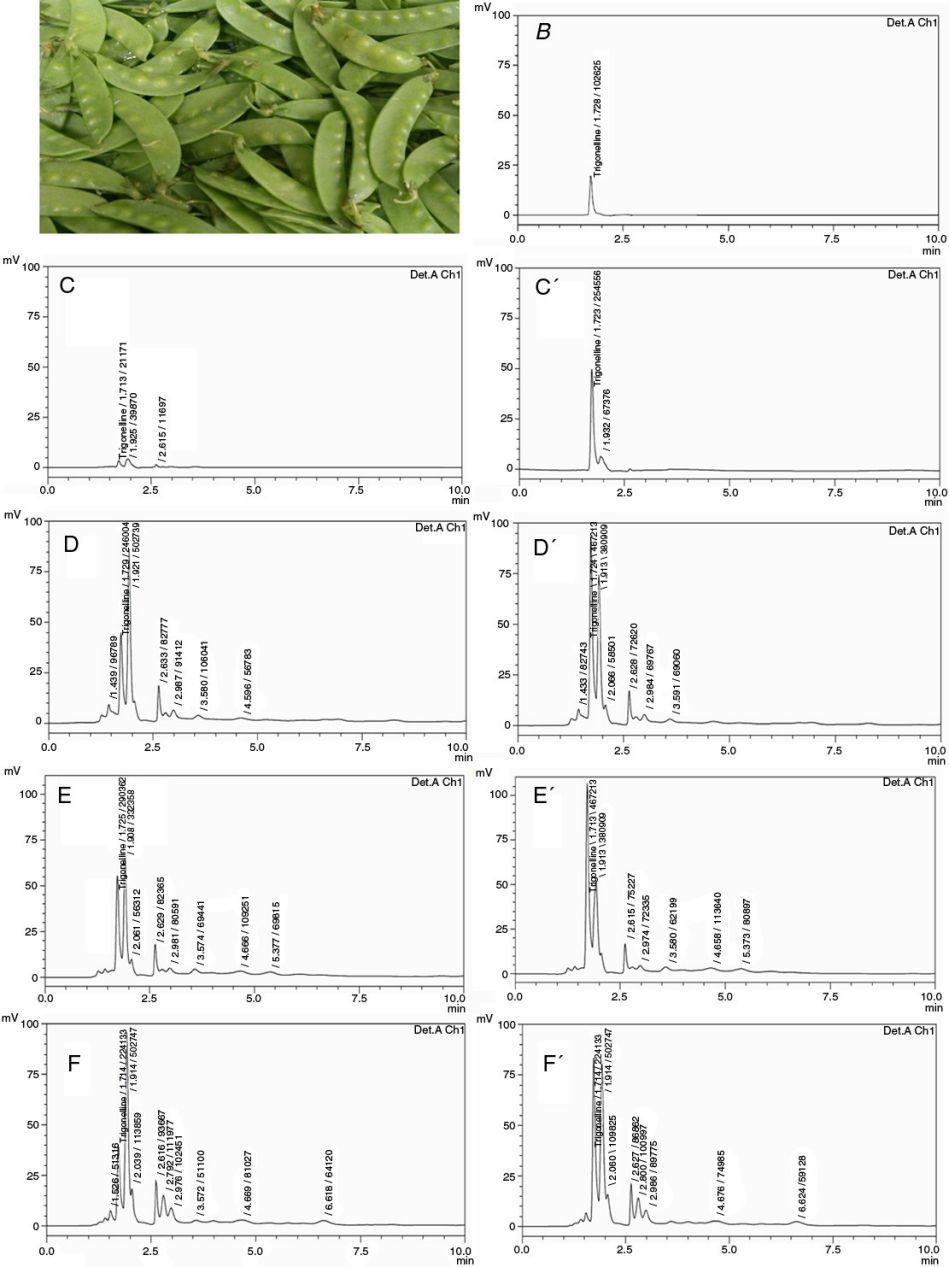
The trigonelline content in *P. sativum* was analyzed by HPLC. (A) Snow pea used in this study was purchased from a traditional market in December in Taiwan Taichung city. (B) Pure trigonelline (5 µg/ml) showed a retention time of 1.728 min. HPLC analysis of the *n*-hexane (C), methanol (D), *n*-butanol (E), and water (F) soluble fractions of *P. sativum* exhibited one peak about at 1.72 min, which was merged with that of trigonelline standard. For qualitative analysis of trigonelline in the fractions of *P. sativum*, 15 µg/ml trigonelline was added to the four fractions of *P. sativum* and then analyzed by HPLC (C′, D′, E′, and F′). Results are representative of three independent experiments.

This study analyzed the trigonelline content in a very popular and versatile Chinese vegetable, snow pea, as a representative proof to prove that trigonelline exists widely in our life. To demonstrate the amount of trigonelline contained in snow pea (Fig. 1A), HPLC was used. Pure trigonelline showed a retention time of 1.728 min (Fig. 1B). HPLC analysis of the four fractions of *P. sativum* exhibited one peak about at 1.72 min (Fig. 1C–F), which was merged with that of trigonelline standard (Fig. 1C′–F′). According to the HPLC data, snow pea has relatively high content of trigonelline. The trigonelline content in *n*-hexane, methanol, *n*-butanol, and water soluble fractions of snow pea is approximately 0.374±0.001, 1.701±0.076, 1.936±0.065, and 1.524±0.021 µg/mg, respectively.

### The effect of trigonelline on cell proliferation of Hep3B cells

**Fig. 2:**
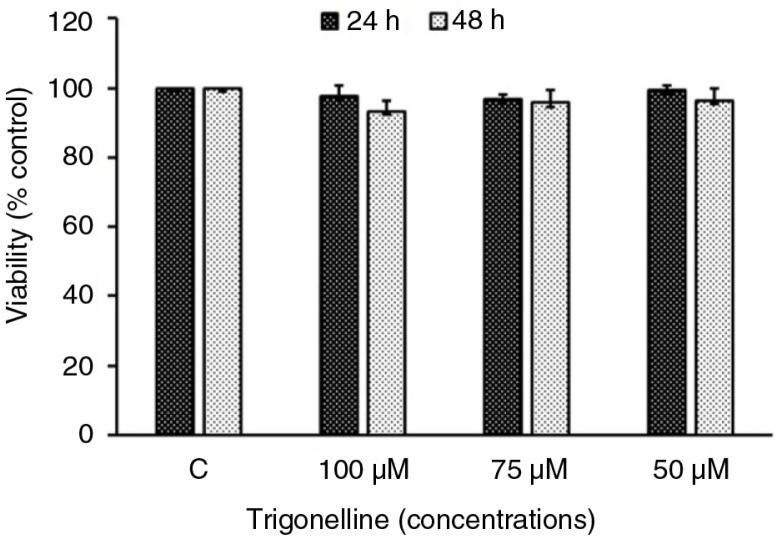
Evaluation of cytotoxicity after incubation of Hep3B cells with trigonelline. Cells were incubated with vehicle alone or with 50, 75, or 100 µM trigonelline for 24 and 48 h. After incubation, the viable cells were measured by MTT assay. The data are presented as proportional viability (%) by comparing the treated group with the untreated group, the viability of which was assumed to be 100%. All results are expressed as the mean percentage of control ±SD of triplicate determinations from four independent experiments.

**Fig. 3:**
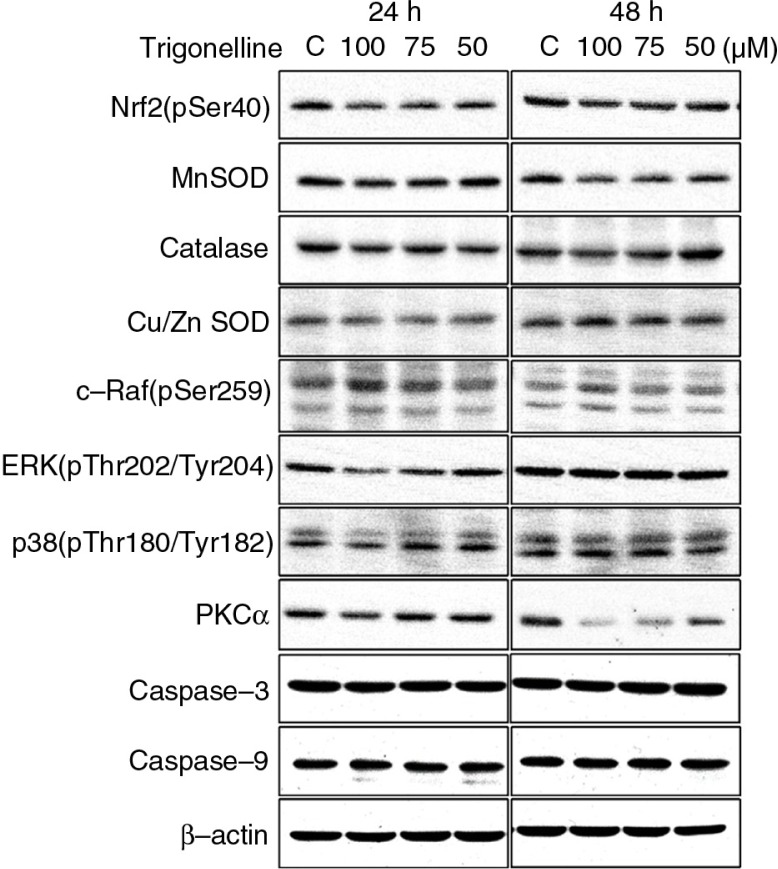
The effects of trigonelline on the protein levels of Nrf2 (pSer40), Nrf2 upstream kinases, and Nrf2-regulated detoxification genes in Hep3B cells. The effects of trigonelline on the protein levels of PKCα, c-Raf (pSer259), ERK (pThr202/Tyr204), p38 (pThr180/Tyr182), Nrf2 (pSer40), catalase, Cu/Zn-SOD, and Mn-SOD were detected by western blot analysis. Cells were incubated with vehicle alone or with 50, 75, or 100 µM trigonelline for 24 and 48 h. Cell lysates were subjected to SDS–PAGE [6% for Nrf2 (pSer40); 7% for PKCα; 8% for c-Raf (pSer259); 10% for catalase; 12% for ERK (pThr202/Tyr204), and p38 (pThr180/Tyr182); 13% for caspase-3, caspase-9, and Mn-SOD; and 15% for Cu/Zn-SOD], and then probed with primary antibodies as described in Materials and Methods section. Results are representative of three independent experiments.

To elucidate whether trigonelline affects the Hep3B cell growth, MTT assay was used in this study. After Hep3B cells were treated with 50, 75, or 100 µM trigonelline for 24 and 48 h, there was no significant difference in cell numbers between control and trigonelline-treated cells (Fig. 2). This study also examined whether trigonelline induced changes of the progression of cell cycle, flow cytometric analysis was performed. After cells were treated with various indicated concentrations of trigonelline for 24 and 48 h, trigonelline had no effect on the cell-cycle distribution of Hep3B cells (Table 1). Based on the above data, MTT assay and cell-cycle analysis did not show any significant difference in Hep3B cell viability and cell-cycle distribution between the control and trigonelline-treated groups, suggesting that trigonelline is not cytotoxic to Hep3B cells. This study also demonstrated that trigonelline had no significant effect on the apoptotic characteristics after 24 or 48 h of treatment. After treatment with trigonelline, the immunostaining patterns of proform caspase-3 and -9 were similar to those seen in control cells (Fig. 3).

**Table 1:** Effects of trigonelline on cell-cycle distribution of Hep3B cells

	24 h			48 h		
		
Trigonelline	G0/G1	S	G2/M	G0/G1	S	G2/M
Control	72.37±8.77	16.05±6.86	11.58±2.04	77.40±0.47	9.78±1.70	12.81±1.43
100 µM	72.10±7.31	15.07±4.18	12.83±3.15	77.58±1.56	10.57±2.36	11.85±0.88
75 µM	72.96±8.46	14.09±4.05	12.96±4.57	77.44±0.74	10.38±0.45	12.18±1.15
50 µM	71.41±7.32	16.89±2.60	11.69±5.67	77.39±0.80	11.40±2.25	11.21±1.93

Hep3B cells were treated with vehicle alone or with 50, 75, or 100 µM trigonelline for 24 and 48 h. After treatment, cells were stained with propidium iodide and subjected to cytometric analysis. The percentages of each phase in the cell cycle are expressed as mean ±SD of three independent experiments.

### The effect of trigonelline on the migration potential of Hep3B cells

**Fig. 4:**
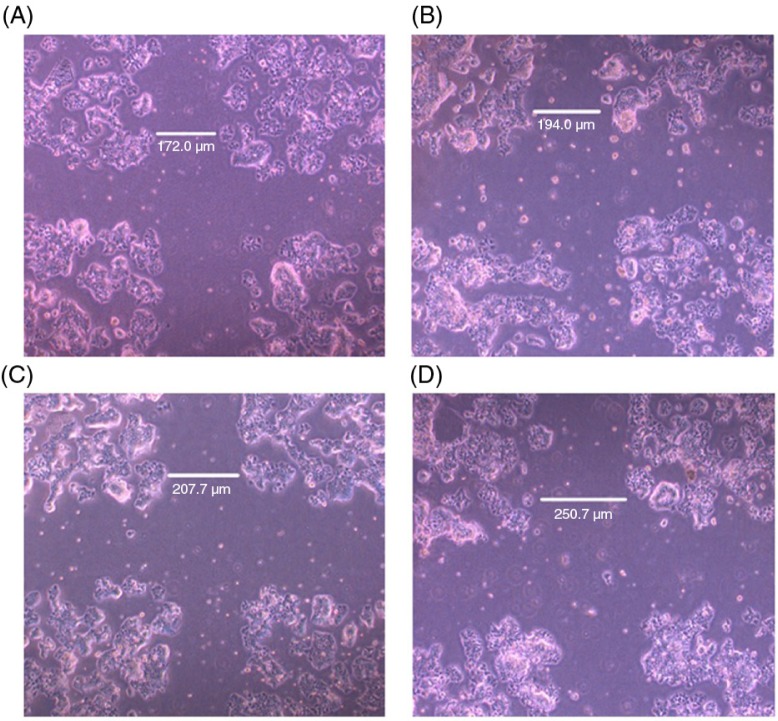
Effect of trigonelline on the migration of Hep3B cells as revealed by the wound assay. Hep3B cells were wounded as described in the Materials and Methods section. After washing, fresh culture medium, containing vehicle alone or various concentrations of trigonelline, was added. Photographs were taken after 48 h of incubation in the absence (A) or the presence (B–D) of trigonelline. Concentrations of trigonelline: 50 µM (B), 75 µM (C), and 100 µM (D). Results are representative of three independent experiments.

Results described above indicated that trigonelline showed no effect on the cell proliferation and progression of cell cycle. Controlling cancer cell invasion and metastasis has been considered to lead to the development of novel strategies in cancer prevention and therapy. This study further examined the effect of trigonelline on anti-invasive activity of Hep3B cells. Since cancer cell migration is a key feature for tumor cell invasion and metastasis, a wound-healing assay was performed to determine whether trigonelline can inhibit Hep3B cell migration. Results of the ‘wound-healing’ assay in vitro showed that in untreated cultures the cells on the edges of the artificial wound migrate toward the wound area within 48 h, while in trigonelline-treated cultures cell migration and motility was inhibited in a dose-dependent manner (Fig. 4). In this study, the cell migration speed significantly decreased with the increase in the distance from the wound edge after treatment with trigonelline (Fig. 4). The distance from the wound edge of control, 50, 75, and 100 µM trigonelline is 178.1±7.6, 196.7±7.3, 208.8±14.0, and 244.3±13.6 µm, respectively.

### The effect of trigonelline on the gene expression of MMP-2, -7, and -9 in Hep3B cells

**Fig. 5:**
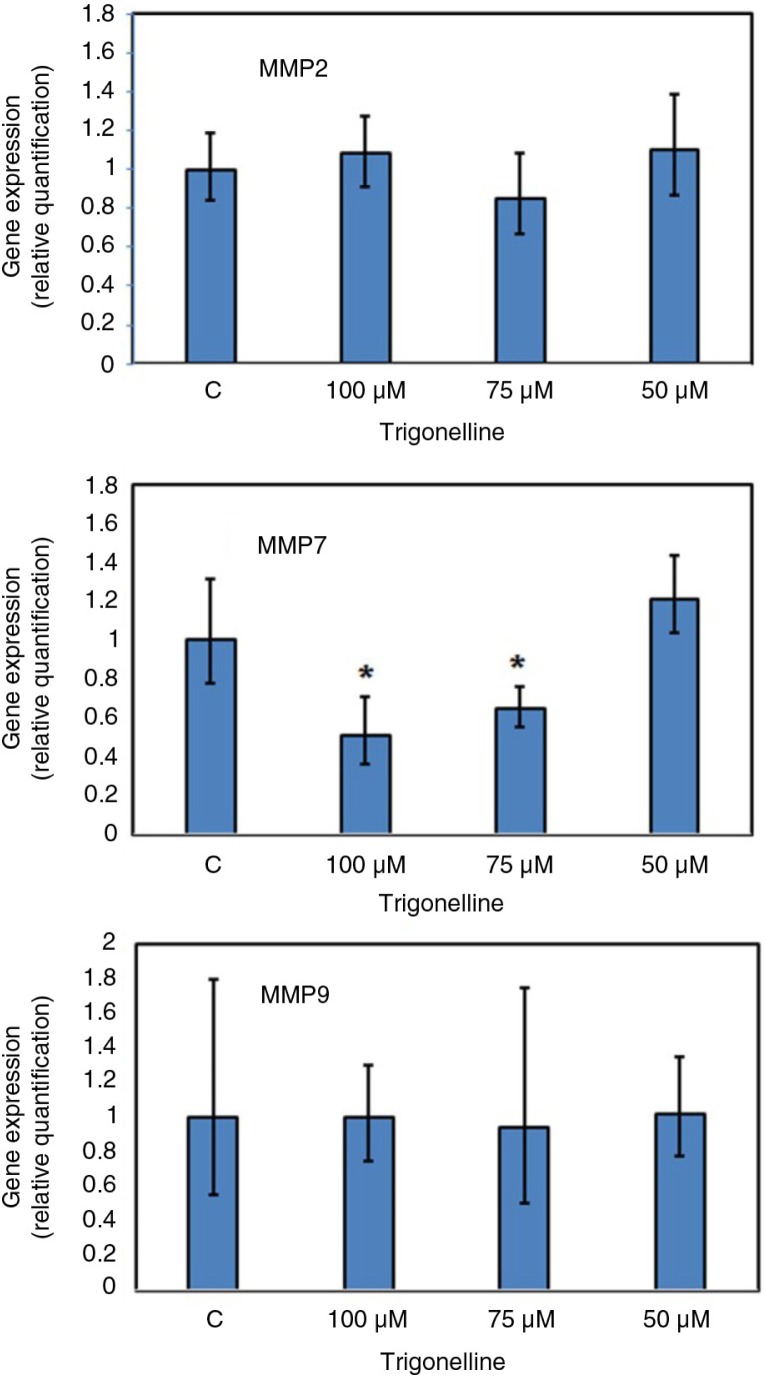
Effects of trigonelline on the mRNA expression of MMP-2, -7, and -9 in Hep3B cells. Trigonelline-induced gene expression of MMP-2, -7, and -9 was detected by real-time RT-PCR. Hep3B cells were incubated with vehicle alone or with 50, 75, or 100 µM trigonelline for 24 h. RNA samples were prepared from control and trigonelline-treated cells. Results were expressed as fold changes and were normalized to GAPDH. Data are represented as mean±SD. *p<0.05 compared to the control values. Results are representative of three independent experiments.

MMP-2, -7, and -9 are thought to be important in tumor metastasis and tissue remodeling; therefore, the present study investigated the gene expression of MMP-2, -7, and -9 during the treatment of Hep3B cells with trigonelline for 24 h. To assess the effect of trigonelline on the mRNA levels of MMP family members, real-time RT-PCR techniques were performed in this study. After Hep3B cells were treated with 75 and 100 µM trigonelline for 24 h, there was a significant decrease in the gene expression of MMP-7 in Hep3B cells (Fig. 5). It is worthy to note that trigonelline had no significant effect on the MMP-2 and -9 gene expression (Fig. 5). Due to trigonelline-induced downregulation of MMP-7 gene expression, we hypothesized that the inhibitory effect of trigonelline on Hep3B cell migration might be associated with MMP-7 activity.

### The effect of trigonelline on the protein expression of Nrf2 phosphorylated at serine 40 and upstream kinases of Nrf2 in Hep3B cells

Since several recent studies have shown that the activation of Nrf2 is involved in migration and invasion of cancer cells, the protein expression of Nrf2 (pSer40) was detected during treatment of Hep3B cells with trigonelline for 24 and 48 h. As shown by immunoblotting, levels of Nrf2 (pSer40) protein was decreased during 100 µM trigonelline treatment 24 and 48 h (Fig. 3). The present study also elucidated whether the expression of PKCα and Raf/ERK pathway members, upstream kinase of Nrf2, is involved in trigonelline-induced decrease in the expression of Nrf2 (pSer40) in Hep3B cells. By western blotting analysis, PKCα, ERK1/2 (pThr202/Tyr204), and p38 (pThr180/Tyr182) protein levels significantly decreased after treatment with 100 µM of trigonelline for 24 h, but c-Raf (pSer259) increase (Fig. 3). It is well known that phosphorylation of Raf on serine-259 is associated with the inactivation of Raf.

### The effect of trigonelline on the protein expression and activity of Nrf2-dependent anti-oxidative enzymes

**Fig. 6:**
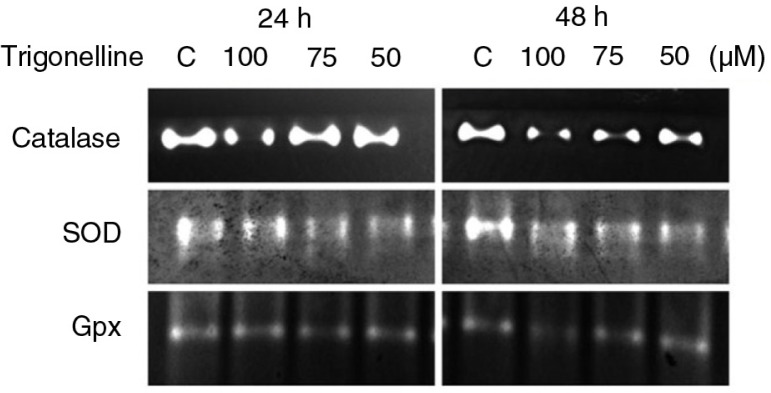
Effects of trigonelline on superoxide dismutase (SOD), catalase, and glutathione peroxidase (Gpx) activity in Hep3B cells. Cells were treated with vehicle alone or with 50, 75, or 100 µM trigonelline for 24 and 48 h. Native gel analysis was performed to analyze the activity of SOD, catalase and glutathione peroxidase. Proteins were separated by electrophoresis through a 10% native PAGE gel. Activities of SOD, catalase, and glutathione peroxidase were analyzed as described in Materials and Methods section. Results are representative of three independent experiments.

Nrf2 might serve as a transcription factor responsible for the induction of detoxifying enzymes, such as SOD and catalase. Our study demonstrated the expression of Cu/Zn-SOD, Mn-SOD, and catalase protein levels during trigonelline treatment 24 and 48 h. The protein levels of Cu/Zn-SOD, Mn-SOD, and catalase were decreased during treatment with 100 µM trigonelline for 24 and 48 h (Fig. 3). This study also examined whether trigonelline induced changes in the activity of SOD, catalase, and glutathione peroxidase. In this study, native gel analysis was performed to analyze the activity of SOD, catalase, and glutathione peroxidase. As illustrated in Fig. 6, exposure of Hep3B cells to 100 µM trigonelline for 24 and 48 h resulted in decreases in SOD, catalase, and glutathione peroxidase activity. These data suggested that Raf/ERK/Nrf2 and further downstream anti-oxidative enzymes are involved in trigonelline-mediated inhibition of Hep3B cell migration.

## Discussion

Cancer chemopreventive agents might prevent the carcinogenic progression during the early stages or premalignant by inducing cancer cell apoptosis (13–15). Natural products, especially dietary food plants, have also received renewed interest in recent years for the discovery of cancer chemopreventive agents. This study analyzed the trigonelline content in a very popular and versatile Chinese vegetable, snow pea, as a representative proof to prove that trigonelline exists widely in our life. The HPLC data showed that snow pea has a relatively high level of trigonelline. The trigonelline content in *n*-hexane, methanol, *n*-butanol, and water soluble fractions of snow pea is approximately 0.374±0.001, 1.701±0.076, 1.936±0.065, and 1.524±0.021 µg/mg, respectively. We also demonstrated that trigonelline had a significant migration inhibition, but not anti-proliferation, of Hep3B cells. Coffee beans have been demonstrated to have an extremely high trigonelline content (2, 3), and coffee consumption has an inverse relationship with the risk of cirrhosis or hepatocellular carcinoma (8, 9). Therefore, our results seemed to indicate that coffee reduced the risk of cirrhosis and/or hepatocellular carcinoma could be the effect of trigonelline on hepatoma cells. Since trigonelline is derived from dietary food plants, it could be a pharmacologically safe natural agent for cancer treatment. Therefore, trigonelline might assert its anti-tumor activity in tumor cell migration, which is necessary at the initiation of the metastatic progression of cancer cells, and develop into a dietary chemopreventive agent.

Nrf2 has a complex role in cancer and has been found to be a key regulator determining cell survival. Recent studies have demonstrated that an increase in Nrf2 activity is implicated in cancer malignancy and chemoresistance. It has been verified that Nrf2 is upregulated in many malignant tumor tissues (17–20), and overexpression of Nrf2 enhances tumor resistance to chemotherapeutic agents in some lung carcinoma, breast adenocarcinoma, and neuroblastoma cell lines (35). Pan et al. (29) also suggested that enhanced expression of Nrf2 promotes glioma cell invasion and migration, whereas reduced expression of Nrf2 attenuates it. However, Nrf2 has been viewed as a ‘good’ protein that protects humans from genotoxic damage caused by carcinogens. Many chemopreventive compounds exert their chemopreventive activity by inducing the Nrf2-dependent response, including phase II detoxifying enzymes and antioxidants that defend cells from oxidative damage (13–15). Boettler et al. (6) have indicated that coffee constituents can modulate the nuclear translocation of Nrf2 and expression of Nrf2 downstream antioxidant genes. Furthermore, the activation of Nrf2, phosphorylation at serine 40, and further downstream antioxidant genes have been proven to be involved in cancer prevention (14, 15). According to the results of western blotting analysis, we demonstrated that trigonelline has an inhibitory effect on Nrf2 by decreasing the phosphorylation of Nrf2 at serine 40 that might result in inactivation of Nrf2. These data suggested that the inhibition of Nrf2 phosphorylation by trigonelline was involved in trigonelline-mediated Hep3B cells migration inhibition.

Nrf2 has been shown to regulate the expression of many antioxidant enzymes such as glutathione peroxidases, SOD, catalase, and heme oxygenase-1 (11, 12). The present study demonstrated that the activity of SOD, catalase and glutathione peroxidase was involved in the inhibition of migration of Hep3B cells by trigonelline. This result is consistent with the previous observation in which trigonelline efficiently suppressed Nrf2 activity accompanied with a decrease in downstream genes activity (36). The potential mechanisms of Nrf2 phosphorylation by antioxidants have also been reported as a function of signaling by p38 mitogen-activated kinase, PKC, and extracellular signal-regulated kinases (ERK) (21–24). In this study, trigonelline (100 µM, 24 h)-induced decrease in PKCα, ERK1/2 (pThr202/Tyr204), and p38 (pThr180/Tyr182) protein levels was observed. However, the protein expression of c-Raf (pSer259) increased after trigonelline treatment. Phosphorylation of Raf on serine-259 has been demonstrated to be associated with the inactivation of Raf (37). The present study demonstrated that PKCα, c-Raf, ERK, and p38 might be an upstream activator of Nrf2 and regulated the Nrf2 activity during trigonelline-mediated inhibition of Hep3B cell migration. Here, we discovered the role of antioxidant enzymes, including glutathione peroxidase, catalase, and SOD, in inhibiting the migration of hepatoma cancer cells after treatment with trigonelline. Based on the above data, trigonelline exposed Hep3B cells inhibited Nrf2 activation and consequently downregulated the expression of cell antioxidant machinery, including SOD, catalase, and glutathione peroxidase expression, through the involvement of PKCα and Raf/ERK pathway.

In this study, the cell migration speed significantly decreased with the increase in the intermediate area of the repairing cultures after treatment with trigonelline. The MMP-2, -7, and -9 are thought to be associated with tumor invasion, metastasis, and angiogenesis. Several recent studies have also shown that Nrf2 is involved in migration and invasion of cancer cells, which may be related to MMP-2 and -9 (29, 30). It is worthy to note that trigonelline had no significant effect on the MMP-2 and -9 gene expression in this study. Although MMP-2 and -9 have been demonstrated to play a central role in cancer metastasis in recent years, the trigonelline regulation of Hep3B cell migration is not mediated by the decrease in MMP-2 and -9 gene expression. MMP-7 has wide proteolytic activity and is capable of activating other MMPs. Thus, it may play several roles during tissue remodelling. In contrast to other MMPs, which are usually expressed in stromal tissue, MMP-7 is expressed mainly on the tumor cell surface (38, 39). With the proteolytic activity, MMP-7 expression has been associated with the potential of cancer cell invasion and lymph node metastasis (39–42). It has been suggested that a positive relationship exists between MMP-7 expression and the invasive potential of cancer cells (42). The levels of MMP-7 in sera or saliva have been found to be used as a diagnostic and prognostic marker of patients with colorectal cancer and oral squamous cell carcinoma (43, 44). These results suggested that the expression of MMP-7 may be closely related to the occurrence and progression of cancer. In addition to extracellular matrix degradation, MMP-7 enhances tumor progression by inhibiting apoptosis and reducing cell adhesion of cancer cells (45, 46). Our data clearly demonstrated that the gene expression of MMP-7 was downregulated by trigonelline, which mediates Hep3B cells migration inhibition through inhibiting the Raf/ERK/Nrf2 signaling pathway. This result is consistent with the previous observations in which activation of ERK signaling in pancreatic cancer was demonstrated to enhance MMP-7 activity (45, 46).

## Conclusions

In this study, trigonelline was demonstrated to have a significant inhibition effect on the migration of Hep3B cells. PKCα and Raf/ERK/Nrf2 signaling pathway and MMP-7 gene expression might play a key role in trigonelline-mediated migration inhibition of Hep3B cells. Trigonelline-induced migration inhibition might be related to its ability to change the activity of SOD, catalase, and glutathione peroxidase of Hep3B cells. This study analyzed the trigonelline content in a popular vegetable, snow pea, as a representative proof to prove that trigonelline is often found in the daily intake of food. Therefore, the results of the present study might regard trigonelline as an attractive chemopreventive and chemotherapeutic agent derived from natural sources in liver cancers.
